# Supplementary material for: Causes and consequences of sex-chromosome turnovers in Diptera
Source: Evol Lett. 2026 Mar 12;10(3):253–68. doi: 10.1093/evlett/qrag003 (PMC13231274; doi:10.1093/evlett/qrag003)
Supplement: qrag003_Supplemental_Files [file qrag003_supplemental_files.zip › Supplementary_Datasets.zip!/Supplementary_Datasets/Dataset_2_Graphical_representation_outgroups/Calliphoridae_Calliphora_vomitoria_chromosomes.pdf]

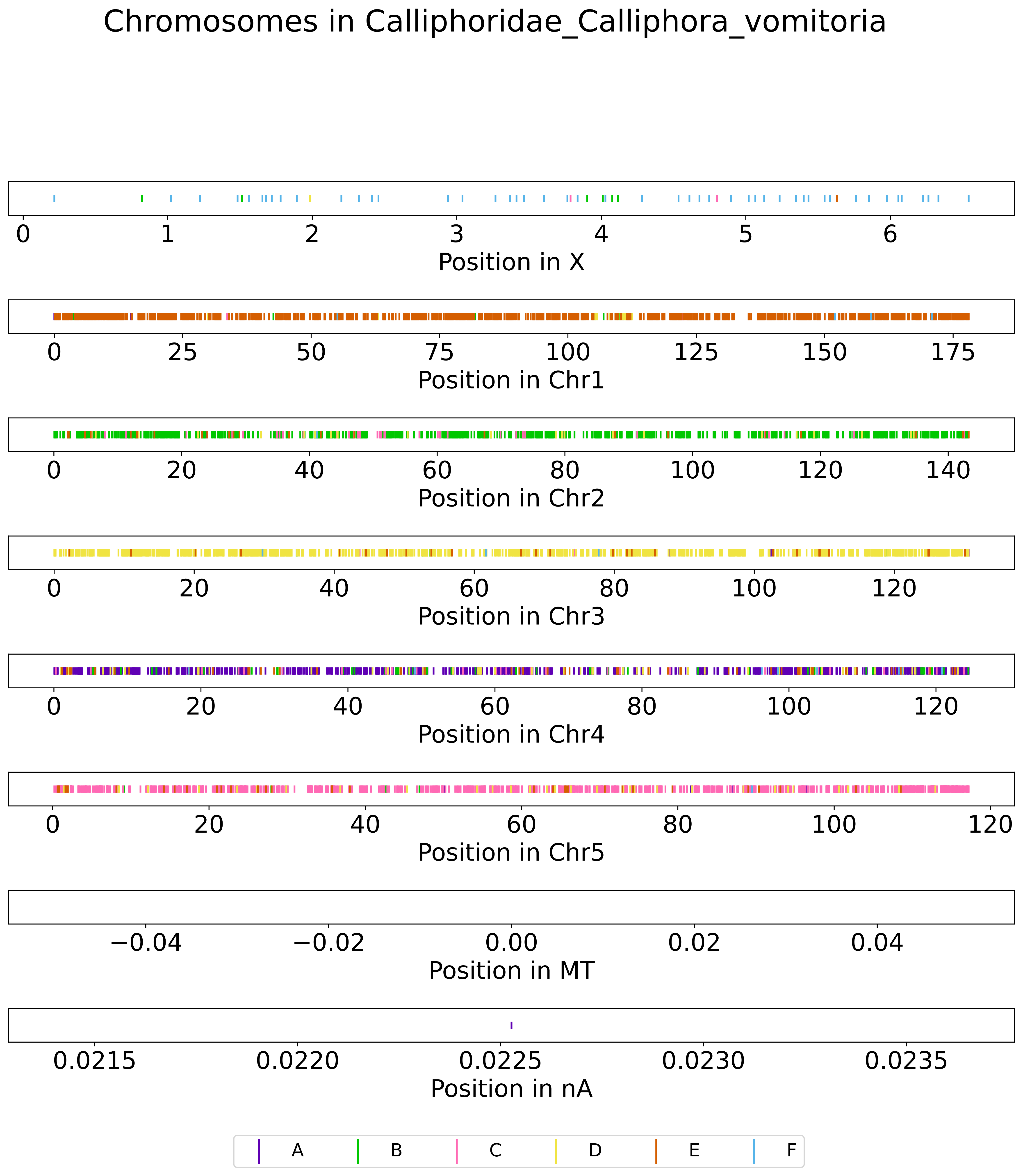

Chromosomes in Calliphoridae_Calliphora_vomitoria
0
1
2
3
4
5
6
Position in X
0
25
50
75
100
125
150
175
Position in Chr1
0
20
40
60
80
100
120
140
Position in Chr2
0
20
40
60
80
100
120
Position in Chr3
0
20
40
60
80
100
120
Position in Chr4
0
20
40
60
80
100
120
Position in Chr5
−0.04
−0.02
0.00
0.02
0.04
Position in MT
0.0215
0.0220
0.0225
0.0230
0.0235
Position in nA
A
B
C
D
E
F
